# A study on the correlation between serum anti-Müllerian hormone levels in male participants of pre-pregnancy genetic health checkup in Qingyang District, Chengdu City

**DOI:** 10.3389/fendo.2026.1931330

**Published:** 2026-10-27

**Authors:** Huafang Kou, Chengyan Gui, Fang Liu, Xiangyue Sheng

**Affiliations:** Department of Clinical Laboratory, Chengdu Qingyang District Maternal and Child Health Hospital, Chengdu, Sichuan, China

**Keywords:** anti-Müllerian hormone, correlation study, male fertility, pre-pregnancy prenatal health examination, semen quality

## Abstract

**Objective:**

To explore the relationship between serum anti-Müllerian hormone (AMH) levels and semen quality in men undergoing preconception health checkups.

**Methods:**

A total of 145 male preconception patients were enrolled. Semen parameters, including sperm kinetics, were measured via computer-aided sperm analysis (CASA), and serum AMH levels were detected using chemiluminescence immunoassay. Spearman’s correlation and multivariate linear regression analyses were used for statistical analysis.

**Results:**

A total of 145 men were included in the study. The median serum AMH level was 8.79 ng/ml, with an interquartile range of (5.25- 11.25) ng/mL. Spearman’s rank correlation analysis showed that serum AMH levels were positively correlated with total sperm count (*r* = 0.268, *P* = 0.001), sperm concentration (*r* = 0.219, *P* = 0.008), total PR count (*r* = 0.225, *P* = 0.006), VCL (*r* = 0.196, *P* = 0.018), VSL (*r* = 0.179, *P* = 0.032), VAP (*r* = 0.164, *P* = 0.049), and STR (*r* = 0.165, *P* = 0.047). Multivariate linear regression analysis showed that after adjusting for confounding factors, such as age and abstinence days, serum AMH remained independently associated with total sperm count (*β* = 0.549, *P*< 0.001), total PR count (*β* = 0.584, *P*< 0.001), and VCL (*β* = 0.572, *P* = 0.005).

**Conclusion:**

Serum AMH levels in men showed weak but statistically significant positive correlations with total sperm count (r = 0.268), total PR count (r = 0.225), and selected sperm kinetic parameters (r = 0.164–0.196). After adjusting for confounders, AMH remained independently associated with total sperm count, total PR count, and VCL, although the overall explanatory power was limited. These findings suggest that serum AMH may have a limited role as an adjunctive biomarker in male fertility assessment; however, its clinical utility is uncertain, given the weak strength of the associations. Large-scale prospective studies are required to validate these findings and determine whether AMH provides incremental value beyond conventional semen analysis.

## 1 Introduction

In recent years, a continuous decline in semen quality among men worldwide has been documented, with male factors accounting for nearly 50% of infertility cases (1). Semen analysis, the cornerstone diagnostic tool for evaluating male fertility potential, provides critical laboratory data, including sperm concentration, motility, and morphology. However, semen parameters are susceptible to various preanalytical and analytical variables, such as abstinence duration, collection conditions, and laboratory techniques, and exhibit considerable intraindividual variability, limiting their capacity to fully reflect the true state of testicular spermatogenic function (2). Consequently, the identification of stable, objective, and quantifiable serum biomarkers has emerged as a research priority in the field of male reproductive medicine.

Anti-Müllerian hormone (AMH), a member of the transforming growth factor-β superfamily, is constitutively secreted by Sertoli cells (supporting cells) in the testes from fetal life to adulthood (3). During male fetal development, AMH mediates the regression of the paramesonephric (Müllerian) ducts, which is a critical step in male sexual differentiation. Postnatally, the continued secretion of AMH by Sertoli cells directly reflects the total Sertoli cell population and functional status (4). Given that each Sertoli cell can support only a finite number of germ cells during spermatogenesis, serum AMH has been proposed as an indirect, but stable, indicator of testicular spermatogenic capacity.

In recent years, a growing body of research has demonstrated positive associations between circulating AMH concentrations and key male reproductive parameters, including testicular volume, total sperm count, and sperm concentration (5). These findings support the concept that AMH may serve as a surrogate marker for the Sertoli cell pool, which, in turn, limits the quantitative output of spermatogenesis. However, other studies have reached divergent conclusions, suggesting that AMH primarily reflects Sertoli cell maturation status rather than spermatogenesis intensity (6). Moreover, the relationship between AMH and sperm motility parameters, such as curvilinear velocity (VCL), straight-line velocity (VSL), and average path velocity (VAP), remains inadequately characterized, and existing evidence is conflicting.

Importantly, most available studies have focused on infertile populations or men presenting to andrology clinics, with relatively few large-scale investigations targeting healthy men undergoing routine preconception health checkups (7). Furthermore, substantial heterogeneity exists across studies regarding assay methods, age ranges, and adjustment for confounding factors such as abstinence duration, which limits the generalizability of the current conclusions. Chengdu, a major metropolitan center in southwestern China, has implemented widespread preconception health screening programs, providing an ideal platform for male fertility assessment. However, serum AMH has not yet been incorporated into routine clinical practice in this setting, and its relationship with comprehensive semen parameters, including both conventional sperm counts and kinematic parameters, in this specific population remains unknown.

To address these gaps, the present study was conducted using data from 145 men who underwent preconception health check-ups at the Maternal and Child Health Hospital in the Qingyang District of Chengdu. We aimed to analyze the correlation between serum AMH levels and both routine semen and sperm kinetic parameters and to evaluate the potential clinical utility of AMH as an auxiliary indicator for male fertility assessment, thereby providing evidence to inform the optimization of preconception health check-up protocols.

## 2 Data and methods

### 2.1 Research object

A total of 145 males who underwent pre-pregnancy health checkups at the Maternal and Child Health Care and Family Planning Service Center in Qingyang District, Chengdu City, from April 2025 to October 2025, were selected as the research subjects. The inclusion criteria were as follows: (1) age 20–45 years; this age range was selected because (a) it encompasses the typical reproductive age range defined by the World Health Organization and is widely used in male fertility studies (8, 9), and (b) men older than 45 years have significantly lower fertility intentions and are more likely to have chronic comorbidities that may confound the assessment of reproductive function (10); (2) voluntary participation in the study and provision of written informed consent; (3) no serious diseases affecting vital organs, such as the heart, liver, and kidneys; (4) no congenital abnormalities, infections, trauma, or history of surgery involving reproductive function; and (5) no use of medications affecting reproductive function and no receipt of radiotherapy or chemotherapy within the preceding 3 months. The exclusion criteria were as follows: (1)incomplete collection of semen specimens; (2) severe oligospermia (sperm concentration< 5×10^6^/mL) or azoospermia; (3) hemolytic specimens (free hemoglobin > 1 g/L) orlipemic specimens (triglycerides > 5.6 mmol/L); and (4) semen liquefaction time > 60 min, or abnormal semen viscosity. This study was approved by the hospital’s ethics committee.

The exclusion criteria were severe oligospermia (sperm concentration< 5 × 10^6^/mL) and azoospermia. The rationale for excluding these conditions was two-fold. First, the primary objective of this study was to investigate the correlation between AMH and semen quality across the spectrum of normal-to-moderately impaired spermatogenesis rather than in the context of severe testicular failure. Second, in cases of severe oligospermia or azoospermia, the underlying etiologies are heterogeneous (e.g., obstructive vs. non-obstructive, genetic vs. acquired), and including these cases would introduce substantial confounding factors that could not be adequately addressed given the limited sample size. However, we acknowledge that this exclusion criterion limits the generalizability of our findings to the full spectrum of male infertility, and our results cannot be extrapolated to men with severe spermatogenic impairment. Future studies targeting this high-risk population are warranted.

### 2.2 Methods

#### 2.2.1 Collection and analysis of semen specimens

After all participants had abstained from sexual activity for 2–10 days, the entire ejaculate was collected by masturbation and in a sterile, dry, disposable semen collection cup. The 2- to 10-day abstinence window was chosen based on the WHO laboratory manual recommendation of 2–7 days (8), with the upper limit extended to 10 days to accommodate practical clinical circumstances and maintain consistency with previous large-scale studies in the Chinese population (11). The specimen was immediately sent for examination. After the semen specimens were liquefied in a 37°C water bath, all semen specimens were assayed using a computer-aided sperm analysis system (CASA; model SSA_II sperm analysis system (Suijia, Beijing), in strict compliance with the standardized protocols specified in the 6th edition of the WHO Laboratory Manual for the Examination and Processing of Human Semen (8). The detection parameters included semen volume, pH, total sperm count, sperm concentration, forward motility rate (PR), non-forward motility rate (NP), immobile sperm rate (IM), sperm motility (PR + NP), and sperm motility dynamics parameters [curved velocity (VCL), straight velocity (VSL), average path velocity (VAP), forward direction (STR), beating frequency (BCF), and lateral swing amplitude (ALH)].

Information on nocturnal emissions during the abstinence period was not systematically collected. This represents a potential limitation, as unrecognized nocturnal emissions could affect semen parameters and introduce confounding factors.

#### 2.2.2 Blood sample collection and AMH testing

All participants provided 3 mL of venous blood on the day of semen collection, which was placed in a vacuum blood collection tube without an anticoagulant, left at room temperature for 30 min, centrifuged at 3000 r/min for 10 min to separate the serum, and stored at -20°C for testing. Serum AMH levels were measured using a chemiluminescence immunoassay. The detection kit was purchased from Roche Diagnostics Products (Shanghai), and the detection instrument was an Access 2 Immunoassay Analyzer, Beckman Coulter. All assay procedures were performed in strict compliance with the manufacturer’s protocol, and the intra-assay and inter-assay coefficients of variation were verified to be less than 5% and less than 8%, respectively.

The detection kit was purchased from Roche Diagnostics Products (Shanghai), and the detection instrument was a Roche Cobas 601 automatic chemiluminescence immunoassay analyzer. All assay procedures were performed in strict compliance with the manufacturer’s protocol provided with the kit, and the intra-assay and inter-assay coefficients of variation were verified to be less than 5% and less than 8%, respectively.

Venous blood was collected from all participants on the same day as the semen collection, ensuring temporal concordance between the serum AMH measurement and semen quality assessment. This timing was chosen to minimize potential temporal variability in AMH levels and ensure that the relationship between AMH and semen parameters was not influenced by temporal confounding. Serum AMH assays were performed blinded to the clinical semen results. Laboratory personnel responsible for AMH measurements did not have access to the corresponding semen analysis data at the time of testing, and semen analysis was performed independently by separate laboratory technicians who were unaware of the AMH results. This blinding procedure was implemented to minimize the potential measurement bias.

### 2.3 Statistical analysis

Data were analyzed using SPSS version 26.0. Normally distributed continuous data are presented as the mean ± standard deviation ( $\overset{-}{x}$ ± s) and were analyzed using one-way analysis of variance. When assumption of homogeneity of variance was not met, Welch’s correction was applied. Non-normally distributed continuous data are presented as the median (interquartile range) [M (P_25_, P_75_)] and were analyzed using the Kruskal-Wallis H test. Pairwise comparisons were performed using Dunn’ test with Bonferroni correction. Spearman’s correlation analysis was used to examine the association between serum AMH levels and semen quality parameters. Multivariate linear regression analysis was performed to investigate the association between serum AMH levels and sperm total count, total PR count, and VCL. Before regression analyses, collinearity diagnostics were conducted, with a variance inflation factor (VIF) of< 5, indicating the absence of severe collinearity. P< 0.05 was considered statistically significant.

Sample size calculation: Based on a pilot study involving 30 participants, the correlation coefficient between serum AMH levels and total sperm count was estimated to be r=0.25. With a two-sided significance level of α=0.05 and 80% statistical power, the required sample size was calculated as 123 participants. Considering an anticipated dropout or incomplete data rate of 15%, the target enrollment was set at 145 participants.

For the multivariable linear regression analyses, three dependent variables—total sperm count, total PR count, and VCL—were selected according to the following prespecified criteria: (a) clinical relevance—total sperm count represents the primary measure of spermatogenic output, total PR count is a well-established predictor of fertility outcomes, and VCL is a key kinematic parameter reflecting sperm hyperactivation capacity, which is critical for fertilization; and (b) statistical significance—all three parameters were significantly associated with AMH levels in the Spearman correlation analysis (P<0.05). Although sperm concentration was significantly associated with AMH levels in the correlation analysis, it was not selected because it is a relative measure influenced by semen volume and may be less informative than the total sperm count. Other parameters with borderline statistical significance (VSL, VAP, and STR) were not included to avoid overfitting, given the limited sample size.

For multiple comparisons, the Bonferroni correction was applied to *post-hoc* pairwise comparisons following the Kruskal–Wallis H test. However, no correction for multiple comparisons was applied to the Spearman correlation analyses, which involved 18 comparisons. Applying the Bonferroni correction to these analyses yielded a significance threshold of P<0.0028 (0.05/18). Under this more stringent criterion, only the correlation with the total sperm count (P = 0.001) remained statistically significant. The other six nominally significant correlations—sperm concentration (P = 0.008), total PR count (P = 0.006), VCL (P = 0.018), VSL (P = 0.032), VAP (P = 0.049), and STR (P = 0.047)— were no longer statistically significant. These findings further underscore the weakness of these associations and support the conservative interpretation that the associations between AMH levels and semen parameters are weak and may be partially attributable to the multiple testing. We acknowledge this limitation and have revised our interpretation accordingly in the Discussion section.

The prespecified primary endpoint was the correlation between serum AMH level and total sperm count. The secondary endpoints included the correlations of serum AMH levels with other semen quality parameters, including sperm concentration, total PR count, PR percentage, and sperm kinetic parameters (VCL, VSL, VAP, STR, BCF, and ALH). Total sperm count was selected as the primary endpoint based on its biological plausibility—AMH reflects the number of Sertoli cells, which limits spermatogenic capacity—and its use as the primary outcome in previous seminal studies on AMH and male fertility.

## 3 Results

### 3.1 Characteristics of the study participants

This study included 145 preconception men aged 23–39 years, with an average age of (29.86 ± 3.70) years. The duration of abstinence was 2–10 days, with an average of (4.53 ± 2.17) days. The semen volume ranged from 1.4 to 9.4 mL, with an average of (3.58 ± 1.42) mL. The pH value was 7.2 to 8.0, with an average of (7.51 ± 0.14). All study participants simultaneously underwent routine semen analysis and serum AMH testing.

### 3.2 Distribution of serum AMH levels

The serum AMH levels ranged from 1.95 to >24.00 ng/mL, with a median of 8.79 ng/mL (interquartile range: 5.25–11.25 ng/mL). Based on the quartiles of serum AMH levels, participants were divided into four groups: Q1 (AMH ≤ 5.25 ng/mL, n=37), Q2 (5.25< AMH ≤ 8.79 ng/mL, n=36), Q3 (8.79< AMH ≤ 11.25 ng/mL, n=37), and Q4 (AMH > 11.25 ng/mL, n=35). Quartile-based categorization, a standard approach in epidemiological studies for exploring dose-response relationships, was chosen to facilitate comparisons with previous studies and to enable the detection of non-linear patterns (12).

### 3.3 Comparison of semen quality parameters among different AMH level groups

A comparison of semen quality parameters among the different AMH level groups is shown in **Table 1**. Among them, there were statistically significant differences in the total sperm count, sperm concentration, total PR count, and PR concentration among the different AMH level groups (*P*< 0.05), whereas the differences in the other semen quality parameters were not statistically significant (*P* > 0.05).

**Table 1:** Comparison of semen quality parameters among groups with different serum AMH levels.

Item	Group Q1 (n=37)	Group Q2 (n=36)	Group Q3 (n=37)	Group Q4 (n=35)	F/H value	P value
Age (years)	30.03 ± 3.82	29.72 ± 3.67	29.89 ± 3.75	29.80 ± 3.69	0.065	0.976
Abstinence duration (days)	4.46 ± 2.21	4.58 ± 2.12	4.51 ± 2.24	4.54 ± 2.19	0.022	0.994
Semen volume (ml)	3.42 ± 1.38	3.78 ± 1.47	3.51 ± 1.41	3.59 ± 1.43	0.589	0.623
pH value	7.50 ± 0.15	7.52 ± 0.13	7.51 ± 0.14	7.51 ± 0.14	0.138	0.938
Total sperm count (×10^6^)	326.71 (144.59, 514.92)	266.48 (123.10, 426.57)	353.51 (187.90, 611.85)	428.41 (276.78, 692.33)*#	10.125	0.018
Sperm concentration (×10^6^/ml)	106.93 (49.38, 141.81)	60.85 (28.31, 109.02)*	110.21 (60.33, 142.07)#	99.85 (63.70, 188.96)#	11.367	0.010
Total progressive motile sperm count (×10^6^)	155.02 (48.54, 214.40)	80.76 (28.02, 137.63)*	136.97 (39.24, 245.70)#	182.02 (88.12, 322.76)#	13.892	0.003
Progressive motile sperm concentration (×10^6^/ml)	39.78 (14.66, 60.16)	22.06 (10.57, 36.37)*	38.93 (12.13, 59.66)#	43.23 (22.66, 69.05)#	9.754	0.021
Progressive motility (PR, %)	40.10 (27.99, 50.10)	36.53 (26.77, 42.99)	36.39 (30.58, 48.87)	41.81 (30.91, 50.98)	1.326	0.723
Non-progressive motility (NP, %)	26.87 ± 11.32	27.03 ± 11.28	26.92 ± 11.21	26.95 ± 11.26	0.002	0.999
Immotile sperm (IM, %)	35.97 ± 16.95	36.51 ± 16.87	36.32 ± 16.91	36.18 ± 16.89	0.007	0.997
Total motility (PR+NP, %)	64.03 ± 16.95	63.49 ± 16.87	63.68 ± 16.91	63.82 ± 16.89	0.007	0.997
Curvilinear velocity (VCL, μm/s)	30.78 ± 10.92	30.92 ± 10.85	31.56 ± 10.89	31.79 ± 10.81	0.075	0.971
Straight-line velocity (VSL, μm/s)	14.02 ± 5.71	14.15 ± 5.64	14.37 ± 5.68	14.51 ± 5.62	0.069	0.975
Average path velocity (VAP, μm/s)	20.91 ± 7.42	21.05 ± 7.36	21.32 ± 7.39	21.46 ± 7.33	0.072	0.973
Straightness (STR)	0.44 ± 0.13	0.45 ± 0.13	0.45 ± 0.13	0.45 ± 0.13	0.043	0.992
Beat cross frequency (BCF, Hz)	8.89 ± 2.76	8.92 ± 2.71	9.01 ± 2.74	9.06 ± 2.70	0.036	0.987
Amplitude of lateral head displacement (ALH, μm)	2.44 ± 0.91	2.45 ± 0.88	2.48 ± 0.89	2.51 ± 0.87	0.032	0.981

* F value is the test statistic of one-way analysis of variance (ANOVA), applicable to normally distributed measurement data (age, abstinence duration, semen volume, pH value, NP%, IM%, PR+NP%, and sperm kinetic parameters); H value is the test statistic of Kruskal-Wallis H test, applicable to non-normally distributed measurement data (total sperm count, sperm concentration, total PR count, PR concentration, PR%). *P< 0.05, compared with Group Q1 for the same parameter; #*P*< 0.05, compared with Group Q2 for the same parameter.

### 3.4 Correlation analysis of serum AMH levels with semen quality parameters

Bivariate Spearman rank correlation analysis revealed a positive association between serum AMH concentrations and a series of semen quality indicators, including total sperm count, sperm concentration, progressively motile (PR) sperm count, and four core sperm kinetic parameters: VCL, VSL, VAP, and STR (*P*< 0.05). The correlation coefficients for these associations ranged from 0.164 to 0.268, indicating weak correlations (**Table 2**). No significant correlations were observed between AMH and age, abstinence duration, semen volume, pH, or other sperm motility parameters (*P* > 0.05 for all).

**Table 2:** Correlation analysis between serum AMH levels and semen quality parameters.

Item	*R* value	*P* value
Age	-0.061	0.467
Abstinence duration	0.076	0.364
Semen volume	0.117	0.163
pH value	-0.033	0.692
Total sperm count	0.268	0.001
Sperm concentration	0.219	0.008
Total progressive motile sperm count	0.225	0.006
Progressive motile sperm concentration	0.158	0.058
Progressive motility (PR)	0.049	0.555
Non-progressive motility (NP)	-0.056	0.506
Total motility (PR+NP)	0.067	0.516
Immotile sperm (IM)	-0.006	0.941
Curvilinear velocity (VCL)	0.196	0.018
Straight-line velocity (VSL)	0.179	0.032
Average path velocity (VAP)	0.164	0.049
Straightness (STR)	0.165	0.047
Beat cross frequency (BCF)	0.124	0.136
Amplitude of lateral head displacement (ALH)	0.098	0.240

### 3.5 Multivariate linear regression analysis

To further evaluate whether the observed associations were independent of potential confounders, we performed multivariate linear regression analyses with total sperm count, total PR count, and VCL as the dependent variables and age, abstinence duration, semen volume, pH, and serum AMH as the independent variables. The selection of these three dependent variables was based on their clinical relevance (total sperm count as the primary measure of spermatogenic output, total PR count as a key predictor of fertility, and VCL as a representative kinematic parameter reflecting sperm hyperactivation capacity) and their statistical significance in the univariate Spearman correlation analyses. The results showed that after adjusting for the above confounding factors, serum AMH remained an independent factor associated with total sperm count (β = 0.549, P< 0.001; **Table 3**), total PR count (β = 0.584, P< 0.001; **Table 4**), and VCL (β = 0.572, P = 0.005; **Table 5**). The VIF values for all the independent variables were consistently below 5, indicating no significant multicollinearity.

**Table 3:** Multiple linear regression analysis of total sperm count.

Dependent variable	Independent variable	Unstandardized *β* coefficient	Standard error	Standardized *β* coefficient	*T* value	*P* value
Total sperm count	Age	-13.682	20.367	-0.040	-0.672	0.503
	Abstinence duration	22.714	19.811	0.067	1.147	0.254
	Semen volume	112.140	20.336	0.329	5.514	<0.001
	pH value	-29.935	20.358	-0.088	-1.470	0.144
	AMH	186.863	20.454	0.549	9.136	<0.001

**Table 4:** Multiple linear regression analysis of total progressive motile sperm count.

Dependent variable	Independent variable	Unstandardized *β* coefficient	Standard error	Standardized *β* coefficient	*t* value	*P* value
Total progressive motile sperm count	Age	-3.765	10.106	-0.023	-0.373	0.710
	Abstinence duration	1.911	9.830	0.012	0.194	0.846
	Semen volume	43.869	10.090	0.269	4.348	<0.001
	pH value	-6.182	10.101	-0.038	-0.612	0.542
	AMH	95.407	10.149	0.584	9.401	<0.001

**Table 5:** Multiple linear regression analysis of curvilinear velocity (VCL).

Dependent variable	Independent variable	Unstandardized *β* coefficient	Standard error	Standardized *β* coefficient	*T* value	*P* value
Curvilinear velocity (VCL)	Age	0.214	0.306	0.057	0.698	0.486
	Abstinence duration	0.167	0.456	0.029	0.365	0.715
	Semen volume	1.746	0.586	0.237	2.981	0.003
	pH value	-8.585	7.881	-0.088	-1.089	0.278
	AMH	0.572	0.199	0.230	2.868	0.005

Notably, the standardized regression coefficients for AMH were markedly higher than the corresponding Spearman correlation coefficients for each outcome (total sperm count: standardized β = 0.549 vs. r = 0.268; total PR count: standardized β = 0.584 vs. r = 0.225; VCL: standardized β = 0.230 vs. r = 0.196). It is important to clarify that the standardized regression coefficients (β) and correlation coefficients (r) are not directly comparable metrics. The standardized β represents the expected change in the dependent variable (in standard deviation units) per one standard deviation change in the independent variable, adjusted for all other covariates in the model. In contrast, Spearman’s r reflects the unadjusted bivariate rank correlation. The discrepancy between these values may be explained by several factors: (a) the β coefficients may be inflated due to the relatively small sample size and overfitting (as noted above); (b) the bivariate Spearman correlation is non-parametric and may underestimate linear associations due to the non-normal distribution of variables; and (c) the regression model includes covariate adjustment, which can increase the effect estimate for AMH if the covariates are negatively associated with the outcome but positively associated with AMH. Importantly, the standardized β of 0.549 does not imply that AMH alone explains 30% (0.549²) of the variance in the total sperm count; this would be a misinterpretation. The R² values of the regression models (data not shown) were substantially lower, indicating that the overall model explained only a modest proportion of the variance in outcomes. Thus, the β coefficients should not be overinterpreted as indicating a strong predictive ability.

## 4 Discussion

This study focused on the population undergoing pre-pregnancy prenatal health checkups, aiming to explore the correlation between serum AMH levels and semen quality parameters and to assess its potential value as an auxiliary indicator of male fertility. The results showed that serum AMH levels were positively correlated with the total sperm count, total count of forward-moving sperm, and several sperm motility parameters. Even after adjusting for confounding factors such as age and the number of days of abstinence, AMH remained an independent associated factor for these parameters. These findings suggest that serum AMH levels are closely related to male testicular spermatogenic function and may provide new reference information for fertility assessment in pre-pregnancy prenatal health checkups.

The results of this study revealed that the median level of serum AMH was 8.79 ng/mL, and the interquartile range was (5.25, 11.25) ng/mL. This range was largely consistent with the reported serum AMH levels in healthy men (13). Circulating AMH concentrations were positively associated with total sperm count, and the median total sperm count in the Q4 group (428.41×10^6^) exceeded the corresponding value in the Q2 group (266.48×10^6^). Multivariate linear regression analysis showed that the standardized regression coefficient of AMH on the total sperm count was 0.549, suggesting that AMH is an independent factor associated with sperm production capacity. However, given the cross-sectional design of this study, this association should not be interpreted as a causal relationship. The term “independently associated factor” is used to reflect the observational nature of these findings. Peng et al. (11) found in a study of 726 male outpatients that the correlation coefficient between serum AMH levels and total sperm count was 0.227 (*P*< 0.001), which was consistent with the results of this study. However, the correlation coefficient in this study was slightly lower than that in studies of infertile populations (r = 0.3 - 0.4), which may be related to the fact that the study population was healthy men before pregnancy, and the overall semen quality was better, and the correlation between AMH levels and semen parameters was more significant in the fertility-damaged population (14). In this study, the correlation coefficients between AMH levels and total sperm count, sperm concentration, and sperm motility parameters were all< 0.3, indicating a weak correlation. Although statistically significant, the weak correlation suggests that serum AMH levels have limited explanatory power for semen quality. Its clinical application value as an independent predictor is insufficient, and it may be more suitable to be combined with other indicators (such as inhibin B and FSH) for assessing male fertility potential. AMH is a glycoprotein hormone secreted by Sertoli cells (supporting cells) of the testes. Its expression level is directly related to the number and functional state of Sertoli cells (Sertoli cells). During male fetal development, AMH participates in testicular differentiation and development. After puberty, although AMH expression is negatively correlated with Sertoli cell maturation, it continues to reflect the Sertoli cell population. Since each Sertoli cell can support only a finite number of germ cells during spermatogenesis, serum AMH levels can indirectly reflect the total Sertoli cell pool and, consequently, the testicular spermatogenic capacity. The correlation between AMH and sperm count observed in the present study further supports this mechanistic model. Its expression level is directly related to the number and functional state of the supporting cells (15). During the fetal stage in males, AMH participates in the differentiation and development of the testes. After puberty, the expression level of AMH is negatively correlated with the maturity of supporting cells, but it can still reflect the number of supporting cells present. Since each supporting cell can only support a certain number of spermatogenic cells to mature, the serum AMH level can indirectly reflect the total number of testicular supporting cells and, consequently, the testicular spermatogenic capacity (16). The correlation between AMH levels and sperm count in this study further supports this theory. We acknowledge that the non-monotonic pattern observed in the quartile analysis—where the Q1 (lowest AMH) group unexpectedly showed higher total sperm count, sperm concentration, and PR count than the Q2 group—does not fully support a simple linear interpretation that ‘higher AMH levels correlate with better semen quality.’ This observation is methodologically and biologically important because it tempers the overall positive correlation detected in the continuous variable analyses and suggests that the association is not strictly dose dependent.

Several overlapping factors may have contributed to this inconsistency. First, the restricted range of semen parameters in our normozoospermic cohort created a ‘ceiling effect,’ which reduced the discriminative power of AMH compared to studies in infertile populations, where semen quality showed much wider variance (17). Second, quartile categorization inherently sacrifices individual data resolution (18), and discretizing continuous variables can introduce artificial between-group fluctuations and reduce statistical power, making the Q1–Q2 difference potentially a random artifact rather than a true biological trend. This interpretation is supported by the fact that when AMH was analyzed as a continuous variable, the Spearman and regression analyses consistently showed significant positive associations. Third, there may be genuine biological heterogeneity within the Q1 group, where some individuals have constitutively low AMH expression unrelated to spermatogenic capacity; however, without measurements of FSH, inhibin B, or testicular volume, we cannot objectively distinguish these subtypes (19). Additionally, the slightly larger semen volume in the Q2 group (3.78 mL vs. 3.42 mL in Q1) may have diluted the sperm concentration, although this does not fully account for the differences in the total sperm count.

Importantly, this non-linear pattern does not invalidate the significant positive correlations observed using continuous AMH values; however, it does underscore that the relationship is weak and not deterministic. Consequently, this finding reinforces that serum AMH alone is unlikely to serve as a robust standalone predictor; its clinical utility, if any, should be adjunctive and combined with other biomarkers. Future larger studies should avoid arbitrary categorization and include additional hormonal and imaging parameters to determine whether this non-monotonicity represents a statistical artifact or a genuine biological nuance.

Furthermore, when the Bonferroni correction for multiple comparisons was applied to the Spearman correlation analyses, only the correlation with the total sperm count remained significant (*P*< 0.0028). This finding further weakens the evidence for clinically meaningful associations and emphasizes the need for confirmation in larger, independent cohorts in future studies.

In this study, the sperm concentration was the lowest in the Q2 group (60.85×10^6^/mL), which was significantly lower than that in the other three groups. However, there were no statistically significant differences among the Q1, Q3, and Q4 groups. The main reason for this is that sperm concentration is a relative indicator that is greatly influenced by the volume of semen. In the present study cohort, the median semen volume in the Q2 group (3.78 mL) was marginally greater than the corresponding value measured in the Q1 group (3.42 mL), which lowered the sperm concentration of the Q2 group, whereas the total number of sperm, which reflects the absolute sperm production capacity, was higher in the Q4 group than in the Q1 and Q2 groups. This result suggests that when evaluating male fertility, absolute indicators, such as the total number of sperm, should prioritized over relative indicators, such as sperm concentration.

This study found that serum AMH levels were positively correlated with the total number of PRs, and a clear dose-response relationship was observed. The median number of PRs in the Q4 group (182.02×10^6^) was higher than that in the Q2 group (80.76×10^6^). Pairwise comparisons between groups showed that the number of PRs in the Q2 group was significantly lower than that in the other three groups. Multivariate linear regression analysis revealed that AMH was an independent associated factor for the total number of PRs (*β* = 0.584, *P*< 0.001). Previous studies have shown that the total number of PRs is a more accurate indicator of male fertility than the percentage of PRs (20). Guzick et al. (21) found that when the total number of PR was less than 10×10^6^, the natural conception rate significantly decreased; when the total number of PR was greater than 40×10^6^, the natural conception rate reached a plateau. In this study, the median total number of PR in the Q2 group was 80.76×10^6^, which was higher than the critical value for a significant decrease in the conception rate proposed by Guzick et al., but lower than those of the other three groups (Q1, Q3, and Q4). The median total number of PR in the Q4 group was as high as 182.02×10^6^, which was within the optimal fertility range. This result suggests that serum AMH levels are not only related to the number of sperm produced but are also closely related to the fertilization potential of sperm. No statistically significant difference was observed in PR% among the groups in this study (*P* = 0.723), further confirming the superiority of absolute number indicators in evaluating male fertility. The median proportion of PR was 40.10% in the Q1 group and 41.81% in the Q4 group, and this inter-group difference was not statistically significant. In contrast, the total count of PR sperm in the Q4 group was 1.17-fold that of the Q1 group, with a statistically significant difference in fertility potential between the two subgroups.

In this study, although no significant differences were observed in the sperm kinetic parameters (VCL, VSL, and VAP) among the AMH quartile groups by ANOVA (P > 0.05), both Spearman’s correlation analysis and multivariate linear regression showed significant positive associations. This apparent discrepancy is statistically expected and primarily reflects the fundamental differences between the analytical approaches used. The one-way ANOVA artificially discretized continuous AMH values into four ordinal groups, resulting in the loss of individual-level information and reduced statistical power to detect subtle associations (22). In contrast, correlation and regression analyses preserved the continuous nature of AMH data and were therefore more sensitive to weak signal detection. However, we acknowledge that the emergence of this discrepancy also supports an alternative, more conservative interpretation: that the actual correlation is too weak to maintain statistical significance when the data are grouped into subgroups. The fact that the association only becomes detectable with more sensitive continuous-variable methods—and even then with correlation coefficients consistently below 0.3—strongly suggests that the clinical relevance of this relationship is limited.

In this study, the correlation coefficient between AMH and VCL was 0.196 (*P* = 0.018), which was considered weak. This is consistent with the results of the systematic review by Katsioudi et al. (23), where the correlation between AMH levels and sperm motility parameters was generally weak (typically *r* = 0.1 - 0.2). Sperm motility parameters mainly reflect the motility ability of sperm and are influenced by various factors, including sperm energy metabolism, tail structure, and seminal plasma components (24). AMH may indirectly affect the maturation process and energy metabolism of sperm by influencing the function of supporting cells, thereby having a slight impact on sperm motility (25). Although this effect was masked by intra-group variation in group comparisons, it could still be detected in the continuous variable analysis.

This study also has some limitations: (1) It is a single-center cross-sectional study, which makes it impossible to determine the causal relationship between AMH and semen quality; (2) The sample size is relatively small, resulting in significant heterogeneity in the low AMH group. Quartile grouping showed that some parameters in the Q2 group were slightly lower than those in the Q1 group. Further verification is required with a larger sample size in future studies. Additionally, this study excluded patients with severe oligospermia (sperm concentration< 5×10^6^/mL) and azoospermia, and the research results cannot be extrapolated to this high-risk group; (3) The AMH level in semen was not detected, making it impossible to compare the correlation between serum AMH levels and semen AMH and the predictive value for fertility; (4) Long-term follow-up was not conducted, making it impossible to assess the predictive ability of AMH for natural conception outcomes; (5) Detailed lifestyle and clinical history data were not collected, and residual confounding factors could not be completely excluded. Future multi-center prospective cohort studies are needed to further verify the clinical value of serum AMH as a biomarker for male fertility; (6) We did not collect information on nocturnal emissions during the abstinence period, which may have influenced semen quality parameters and could represent an unmeasured confounding factor; (7) The relatively modest sample size (n = 145) may have limited the statistical power for detecting smaller effect sizes, particularly in subgroup analyses and quartile comparisons, where each group contained only 35–37 participants. Although the quartile grouping approach is useful for descriptive purposes, it results in reduced precision for intergroup comparisons. This limitation is particularly relevant when interpreting the non-monotonic patterns observed, as these may be influenced by random variations within small subgroups. Although our sample size was sufficient for the primary correlational analyses based on our pre-hoc power calculation, the subgroup analyses should be interpreted with caution. Future studies with larger sample sizes are required to validate these findings and enable more robust subgroup analyses.

In this cohort of men in the preconception stage, serum AMH levels were weakly but significantly associated with total sperm count, sperm concentration, total progressively motile sperm count, and several sperm kinetic parameters. AMH remained independently associated with total sperm count, total PR count, and VCL after adjusting for potential confounders. However, given the weak correlation coefficients (all r< 0.3), non-monotonic pattern observed in quartile-based comparisons, and limitations of the cross-sectional design, the clinical utility of serum AMH as a standalone biomarker for male fertility assessment remains uncertain. AMH may be valuable as an adjunctive indicator when combined with other clinical and laboratory parameters; however, routine clinical implementation cannot be recommended based on the current evidence. Large-scale, prospective, multicenter studies with longitudinal follow-ups of fertility outcomes are required to establish the predictive value of serum AMH and to define its role in preconception health assessment.

## Data Availability

The original contributions presented in the study are included in the article/supplementary material. Further inquiries can be directed to the corresponding author.
